# A New Incremental Cycling Cognitive‐Motor Dual‐Task Test to Assess Simultaneous Sustained Attention and Neuromuscular Fatigue in Trained Athletes

**DOI:** 10.1111/sms.70122

**Published:** 2025-09-01

**Authors:** Thomas Goepp, Mark Hayes, Pascal Hot, Thomas Rupp

**Affiliations:** ^1^ Inter‐University Laboratory of Human Movement Sciences, LIBM University Savoie Mont‐Blanc, EA 7424 Chambéry France; ^2^ Environmental Extremes Laboratory, School of Education, Sport and Health Sciences University of Brighton Brighton UK; ^3^ LPNC, CNRS URM 5105, University Grenoble Alpes, University Savoie Mont‐Blanc Grenoble France; ^4^ Institut Universitaire de France France; ^5^ Center for Magnetic Resonance in Biology and Medicine, UMR CNRS 6612, Faculty of Medicine of Marseille Marseille France

**Keywords:** central fatigue, exercise, mental effort, oxygenation, performance

## Abstract

Cognitive performance during cognitive‐motor dual‐task (CMDT) varies with exercise intensity. According to the inverted‐U theory, low‐to‐moderate intensity enhances cognition, but this remains unexplored in trained individuals who may better sustain cognitive performance at high intensities due to improved prefrontal cortex (PFC) homeostasis. Additionally, how sustained attention influences neuromuscular fatigue during whole‐body CMDT is unclear. This study investigated both during incremental cycling using an innovative ergometer. Forty trained adults (30 males/10 females; 28.5 ± 7.4 years, 22.9 ± 2.2 kg·m^−2^) performed an incremental cycling test with a sustained attention Mackworth task. The protocol consisted of 3‐min ramp stages (starting at 1 W·kg^−1^, +0.4 W·kg^−1^ per stage) until “extremely strong” perceived effort (Borg CR_100_, task‐failure TF). At each stage, we assessed: Mackworth score, quadriceps isometric maximal voluntary contraction (IMVC), neuromuscular fatigue (peripheral: twitch force, Pt; central: voluntary activation, VA), PFC oxygenation (NIRS), and mental effort. Data were interpolated at 20%, 40%, 60%, and 80% of TF. Pt decreased linearly (−40.7% ± 15.1%, *p* < 0.001). VA declined from 40%TF (−1.5% ± 0.9%, *p* = 0.003) and worsened at 80%TF (−6.9% ± 2.4%, *p* < 0.001), alongside IMVC (−20.9% ± 8.9%, *p* < 0.001). PFC oxygenation dropped from 60%TF (−7.9% ± 2.2%, *p* < 0.001). Mackworth performance declined only at TF (−11.0% ± 6.7%, *p* < 0.001), associated with ≥ 84% ± 7% HR_max_. Sustained attention did not follow the inverted‐U theory during incremental cycling in trained adults. Cognitive impairments were observed at very strong intensities, in correlation with PFC deoxygenation. Central fatigue emerged early, itself correlated with increased mental effort and cerebral deoxygenation.

## 1 Introduction

Cognitive‐motor dual‐task (CMDT) involves the simultaneous execution of motor and cognitive tasks [1]. CMDT scenarios are commonly observed in occupations, and sports where intense physical exertion combined with high cognitive demand, such as maintaining sustained attention while carrying a load in military operations or during high‐intensity soccer gameplay, may reduce task performance. A leading hypothesis proposes an inverted‐U relationship between exercise intensity and cognitive performance [2, 3]. According to this model, cognitive performance is supposed to peak at moderate exercise intensity (the highest point of the inverted‐U curve), likely due to increased arousal facilitated by neurotransmitter release [4]. However, the decline in cognitive performance at higher intensities, as suggested by the inverted‐U relationship, is debated across trained populations [5].

It is suggested that cognitive performance may be less impaired during high‐intensity exercise when individuals experience lower physiological stress at a given relative workload [5]. The physiological profile associated with trained individuals may help attenuate the typical prefrontal cortex (PFC) deoxygenation observed above the respiratory compensation point during physical exercise [6]. Functional magnetic resonance imaging studies highlighted that PFC networks are highly impacted by physical exercise [7] and cognitive tasks relying on executive functions [8] or attentional fields [9]. These statements suggest that sustained PFC oxygenation could help preserve cognitive performance in a model of neural resources allocation during task execution [10]. However, it is likely that not all cognitive dimensions are equally sensitive to PFC networks [11], although other brain regions may also be modulated by exercise intensity during CMDT situations. This raises the question of how specific cognitive domains are differentially affected by exercise‐induced changes in PFC activity in trained individuals.

The inverted‐U framework has primarily been applied to executive functions, considering higher‐order cognitive processes such as working memory, inhibitory control, cognitive flexibility, planning, reasoning, and problem‐solving [12], which represent some of the most extensively studied domains in neuroscience [13]. These functions are thought to be particularly sensitive to fluctuations in PFC oxygenation, with performance modulations frequently observed during concurrent physical exercise [14]. Attention, as a fundamental cognitive resource underlying executive functioning, has also received increasing interest in recent years. Among its subtypes, sustained attention (SA), defined as the capacity to maintain focus over time, is especially relevant in many sport and occupational contexts [15]. Some authors suggest that SA may be less dependent on PFC activation than executive functions and thus potentially less affected by simultaneous physical effort [11]. As a result, it remains unclear whether SA follows the same inverted‐U pattern across different exercise intensities. In parallel, our recent findings indicate that physically demanding CMDT can modulate the etiology of neuromuscular fatigue, particularly during high‐intensity efforts, suggesting substantial interactions between cognitive load and neuromuscular function efficiency.

Neuromuscular fatigue, defined as the exercise‐induced decline in isometric maximal voluntary contraction (IMVC), stems from both peripheral mechanisms (within the muscle) and central mechanisms (proximal to the neuromuscular junction, including the central nervous system) [16]. Recent literature highlighted that CMDTs possibly modulate neuromuscular fatigue etiology by specifically increasing central fatigue compared to exercising alone, but evidence is scarce and limited to two different cases: at exhaustion after isometric contractions at 15% IMVC with surimposed executive function task [17] or after 15 min of cycling at 80% of maximal heart rate with a surimposed SA task [18]. It remains unknown how neuromuscular function is progressively impacted by exercise intensity across a CMDT that would involve large muscle mass (cycling) and a SA task.

Neuromuscular fatigue and particularly central alterations are known to recover very quickly [19]. Most of the studies involving neuromuscular function assessment after global exercise (e.g., cycling, running) suffer from possibly biased interpretations due to the time needed to move the participants to the isometric knee‐extensor ergometer after completion of exercising (30–180 s) [20]. This time‐delay issue weakens the ability to put in correspondence a given level of cognitive performance and a given amount and etiology of fatigue. However, it can be addressed using an innovative cycle ergometer [21]. For that purpose, a new incremental cycling test involving a surimposed cognitive task with regular neuromuscular fatigue assessments at the end of each cycling stage may be relevant to better understand the interaction between cognitive performance and neuromuscular fatigue development.

The present study aimed to simultaneously explore SA performance, PFC oxygenation, and neuromuscular fatigue etiology during an incremental cycling exercise in trained participants. We hypothesized that SA performance would be enhanced for low‐to‐moderate exercise intensities and slightly impaired close to task failure, alongside significant PFC deoxygenation and exacerbated central fatigue.

## 2 Materials and Methods

### 2.1 Participants

Forty healthy trained adults (COG_EX_ group; 10 women and 30 men; age: 28.5 ± 7.4 years, mass: 72.2 ± 9.5 kg, body mass index: 22.9 ± 2.2 kg·m^−2^, training per week: 8.0 ± 3.1 h) provided written informed consent after being informed of the procedures and the risks associated with the main aims of this study. They belonged to Tier 2 according to the participant classification framework [22] and needed to be involved in at least one sport classified as an endurance activity (e.g., cycling, running), which allowed us to reduce interindividual variability in the number of stages performed during the experimental trial (endurance testing). Our sample size, according to GPower 3.1 software calculation, allows detecting a small effect size of *f* = 0.19 (i.e., $\eta_{p}^{2}$ ~ 0.035), with a power of 95%, for the within interaction of a repeated‐measures ANOVA based on cognition results. Participants refrained from intense or unhabitual physical exercise within the 24 h before testing, avoided any consumption of analgesics, alcohol, caffeine, or energetic beverages on test days, slept for at least 7 h the night before the tests, and consumed their last meal at least 2 h before the tests. We decided to recruit a posteriori 40 additional participants, matched on sex, age, and the training status of the COG_EX_ group (COG_MATCHED_ group; 10 women and 30 men; age: 26.4 ± 6.7 years, mass: 70.3 ± 8.7 kg, body mass index: 22.3 ± 2.0 kg·m^−2^, training per week: 8.4 ± 3.2 h). These participants were engaged in a single session of repetitive cognitive tasks without exercise to check for confounding effects (e.g., learning effect, mental fatigue) in the interpretation of cognitive performance across the experimental session in the COG_EX_ group. The study conformed to the standards of the latest Declaration of Helsinki and was approved by the local research ethics committee (CER‐USMB, 2024–03‐BFRDT).

### 2.2 Experimental Design

Participants performed 2 sessions (familiarization followed by experimental session), interspersed by at least 24 h, on an innovative cycle ergometer [23], allowing neuromuscular fatigue assessments with a short delay (i.e., < 3–5 s) during CMDT.

#### 2.2.1 1st Visit: Familiarization

The first visit was conducted to familiarize the participants with cycling at various levels of perceived effort, using the 100‐points Borg scale (CR_100_ [24]) in a semi‐supine position on the ergometer, as well as with the procedures for neuromuscular function assessment. The participant's position on the innovative cycle ergometer was set to allow pedaling as well as immediate evaluation of isometric contractions of the right leg extensors in the horizontal axis. An individual adjustment of the seating position was carried out during isometric quadriceps contractions, when pedals were locked, with a minimum of 90° and 110° for the right angles of the knee and hip, respectively. This position was maintained during the experimental visit. The participants were also used to complete the Mackworth task (*cf*. Section 3) at rest (2 × 30 trials) and while cycling (1 × 60 trials).

#### 2.2.2 2nd Visit: Experimental Session

**FIGURE 1:**
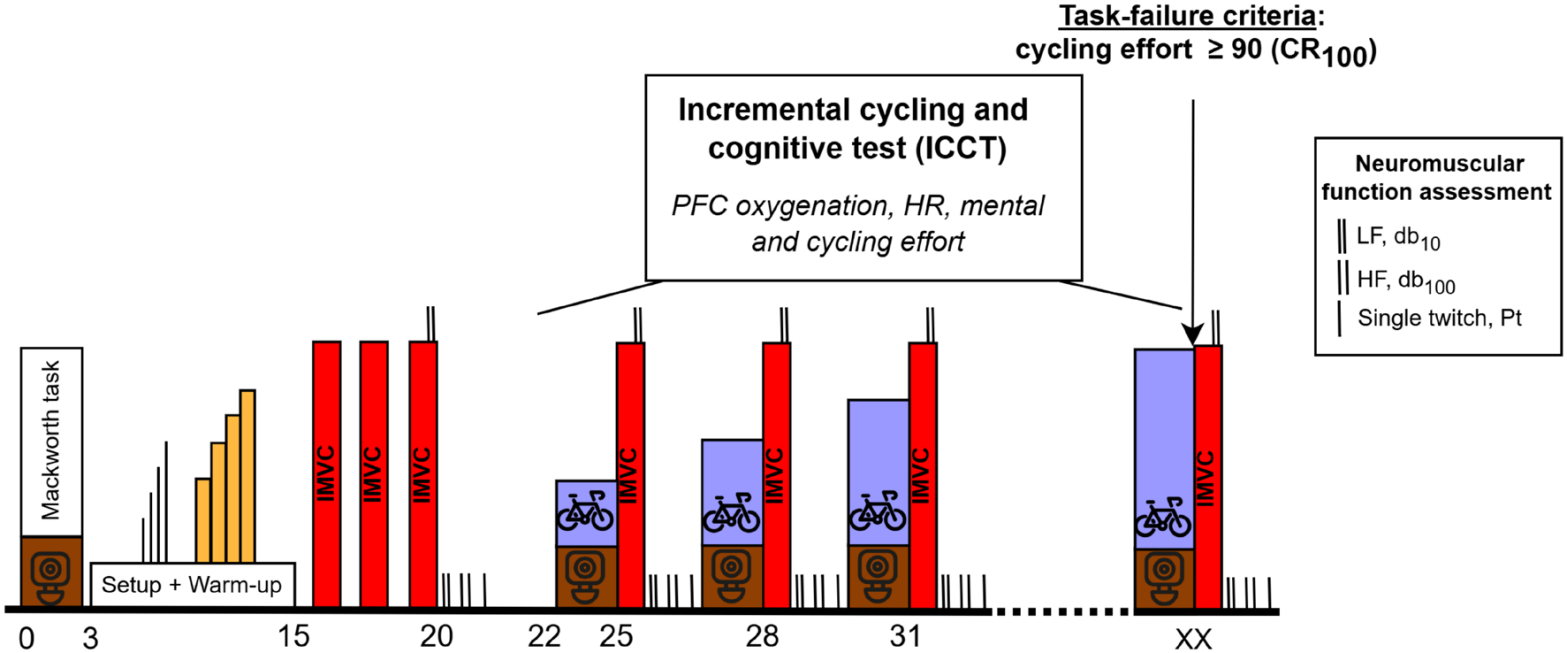
Overview of the experimental session including the incremental cycling cognitive test (ICCT). HF, high‐frequency doublet; HR, heart rate; IMVC, isometric maximal voluntary contraction; LF, low‐frequency doublet; PFC, prefrontal cortex.

After positioning on the ergometer, the participants performed a short practice of the Mackworth task (1 × 30 trials) followed by a 2 min 30 s block of trials at rest. After a baseline evaluation of the knee‐extensor neuromuscular function (see dedicated section below), participants were involved in a new incremental cycling cognitive test (ICCT) on the innovative cycle ergometer. Stages of the ICCT were 3 min at a free cadence ranging between 70 and 90 RPM, and the participants started cycling at 1 watt·kg^−1^ with 0.4 watt·kg^−1^ increments up to a cycling effort of ≥ 90/100 (extremely strong) on the CR_100_. Each cycling stage was combined with the Mackworth task for the first 2 min 30 s, and perceptual responses were assessed during the last 30 s of the stage. PFC oxygenation and heart rate (HR) were continuously recorded, while neuromuscular fatigue was evaluated immediately at the end of each stage. Individual data were extrapolated at 20%, 40%, 60%, and 80% of task failure (TF) to analyze progressive changes across ICCT. This method allowed a relative comparison of participants who completed different numbers of stages before reaching maximality criteria. The experimental session is presented in Figure 1.

### 2.3 Measurements

#### 2.3.1 Cognitive Task

The Mackworth task was employed to evaluate sustained attention (SA) at rest and divided attention during each ICCT stage, using the web‐based platform PsyToolkit [25]. The task involved visualizing a clock‐like dial displaying a green arrow that moved like the second hand of a watch, randomly performing a larger temporal jump (15% of the trials). Participants were instructed to respond to these jumps as quickly as possible by clicking, while ignoring the normal movements of the second hand during the remaining 900 ms intervals when no response was required. Participants were instructed to prioritize both speed and accuracy to mitigate potential trade‐offs between these variables. Reaction time, accuracy, and a composite score that equally weighed these two factors were systematically evaluated. Each block of the task lasted 2 min 30 s, and the three performance indicators were averaged across blocks. Prior to data collection, participants were provided with a comprehensive description of the task and its instructions. At the beginning of the experimental session, participants completed a brief practice block of 30 trials (~30 s) to remind themselves of the task mechanics. A video of a typical Mackworth task sequence is provided in Video S1.

#### 2.3.2 Innovative Ergocycle and Neuromuscular Function Assessments

The ICCT was performed using a cycling ergometer developed in the laboratory to allow immediate and reliable evaluation (< 3–5 s) of the isometric maximal voluntary contraction (IMVC) of the knee‐extensor neuromuscular function at the end of each cycling stage in the semi‐recumbent position [23]. Cycling powers were imposed during the ICCTs from a Wingate Velotron setup (Racermate Inc., Seattle, WA), connected to the ergometer. Cycling power was normalized to body weight and expressed in watt·kg^−1^.

#### 2.3.3 Neuromuscular Function Assessments

To assess peripheral and central indices of neuromuscular fatigue, electrical stimulations were administered percutaneously to the femoral nerve via a cathode electrode (10‐mm diameter; Meditrace 100, Covidien) placed on the inguinal triangle. A rectangular anode electrode (50 × 90‐mm, Durastick Plus, DJO Global, Vista, CA, USA) was attached to the gluteal fold. An electrically induced square wave of 1‐ms duration was delivered using a constant current stimulator (DS7A, Digitimer, Welwyn Garden City, Hertfordshire, UK). At the beginning of each session, to determine the optimal intensity of stimulation, single stimuli were delivered incrementally in steps of 10 mA, every 5 s, until the twitch amplitudes plateaued. The optimal intensity was then increased by 20% for subsequent evaluations to ensure supramaximality. The evaluation started with a 3‐s IMVC with one high‐frequency doublet (Db_100_, HF) evoked during the force plateau. Three electrical stimuli were then delivered to the relaxed muscle interspersed with 3 s between each stimulus: one Db_100_, one low‐frequency doublet (Db_10_, LF), and one singlet (pic twitch, Pt). The ratio of Db_10_ to Db_100_ (LF/HF) was calculated as an index of low‐frequency fatigue [26]. Voluntary activation (VA) corresponding to a central fatigue indicator was calculated using the formula below [27]:

$$\mathrm{VA}\;\left(\%\right)=\left(\;1-\frac{\mathrm{SIT}*\frac{F_{\text{init}}}{\mathrm{MVC}}}{{\mathrm{Db}}_{100}}\;\right)*100$$

where SIT is the amplitude evoked when stimulation was delivered on MVC, *F*_init_ is the initial force during the stimulation, Db_100_ is the amplitude evoked by Db_100_ on relaxed muscle. Neuromuscular evaluation was performed in a non‐fatigued state, before the ICCT (Baseline); immediately at the end of each 3‐min stage and at the end of the ICCT. The duration of the neuromuscular function assessment (interstage delay) was approximately 15 s.

#### 2.3.4 Prefrontal Cortex Oxygenation

PFC oxygenation was continuously monitored during the ICCT using NIRS (Portalite, Artinis Medical Systems, Elst, The Netherlands). The source generated two wavelengths of continuous near‐infrared light (780 and 850 nm), allowing monitoring of changes in oxy‐, deoxy‐, and total hemoglobin concentrations (O_2_Hb, HHb, and tHb respectively). The PFC tissue saturation index (TSI, expressed in %) was calculated by the device based on spatially resolved spectroscopy and was used to assume changes in intracortical oxygen status. The detection probe was positioned over the left PFC area between Fp1 and F3, according to the modified international EEG 10–20 system and firmly secured to the skin with double‐sided tape. A black sweatband was placed over the probe to shield the optodes from the ambient light. NIRS data were collected at 50 Hz, filtered with a 2‐s moving Gaussian smoothing algorithm, and averaged over the first 2 min 30 s of each stage.

A standardized baseline seated position on the cycle ergometer, without moving the legs, was maintained during 2 min just before starting the ICCT. NIRS‐derived hemoglobin concentrations were expressed as relative changes from this baseline.

#### 2.3.5 Heart Rate

HR was monitored throughout the entire experimental session, using a cardiothoracic girdle sensor (Polar E10, Polar Electro 2024, Finland). HR data were averaged over each 3‐min stage and presented as a percentage of maximal HR (%HR_max_) achieved during a field intermittent maximal test (IFT30/15) performed in the 2 weeks before the experiment by each participant (mean HR_max_ of 191.6 ± 8.8 bpm).

#### 2.3.6 Perceived Cycling Effort and Mental Effort

Cycling effort, defined as “the conscious sensation of how hard, heavy, and strenuous exercise is” [28] and mental effort asked as “what is the mental effort associated with the situation?” were assessed during the last 20‐s of each stage across ICCT, using the CR_100_. Once a perceived cycling effort rating of ≥ 90/100 (“extremely strong”) was reported by the participant, the ICCT was stopped, and a last neuromuscular function assessment was performed.

### 2.4 Complementary Experimentation Conducted on a COG_MATCHED_ Group to Get Deeper Insights on Cognitive Function Responses Observed in the COG_EX_ Group

The participants recruited a posteriori (COG_MATCHED_), performed a single experimental session consisting of a repetition of 3‐min blocks of SA task with the exact same parameters and timings as performed during the ICCT for the COG_EX_ group. These participants were familiarized with the task exactly in the same manner as the COG_EX_ participants. The number of blocks realized was matched to the number of blocks performed by the participants of the COG_EX_ group. This was done to identify possible task‐learning effects (potentiation) or task performance deterioration that would be due to mental fatigue or demotivation with the repetitions of the tasks. As for the COG_EX_ group, reaction time, accuracy, and a composite score were evaluated on the Mackworth tasks (see Section 3) and mental effort was assessed during the last 30 s of each 3‐min block. The inter‐block delay was 15 s to mimic ICCT design. The participants were asked to be as efficient as possible on each block and were not informed about the number of SA blocks to be performed to prevent motivational interference and to be placed in the same situation as the COG_EX_ participants. The Mackworth task and mental effort values were analyzed as deltas from resting values to better comprehend the effects of exercise intensity.

For ethical issues related to the main objective of this complementary experimentation, other variables than cognitive responses (e.g., prefrontal oxygenation, neuromuscular responses) were not assessed in the COG_MATCHED_ participants.

### 2.5 Statistical Analysis

Frequential statistics were conducted using JASP software (version 16, Amsterdam, The Netherlands) and statistical significance was set at *p* < 0.05. Data are presented as mean ± SD in the text and in the figures. Shapiro Wilk tests were conducted to assess the normal distribution of the data. In cases where sphericity was violated (Mauchly test), Greenhouse–Geisser corrections were applied. To examine the effects of the ICCT on SA (Mackworth task) and mental effort, two‐way analysis of variance for repeated measures (ANOVAs RM) were performed on 6 time points (main effect of *time* at baseline, 20%, 40%, 60%, 80% and TF) between COG_EX_ and COG_MATCHED_ (main effect of *group*). Secondly, one‐way ANOVAs RM were conducted on NIRS‐derived and neuromuscular function indices on these same 6 time points only for the COG_EX_ group. Power output, %HR_max_, and cycling effort were examined with one‐way ANOVAs RM at 20%, 40%, 60%, 80% and TF. Pairwise Bonferroni procedures were applied for posthoc analyses when ANOVA revealed significant results. The partial eta squared ($\eta_{p}^{2}$) for ANOVA analysis was calculated, where $\eta_{p}^{2}$ < 0.01 indicates a very small effect, 0.01 ≤ $\eta_{p}^{2}$ < 0.06 a small effect, 0.06 ≤ $\eta_{p}^{2}$ < 0.14 a moderate effect, and $\eta_{p}^{2}$ ≥ 0.14 a large effect. For COG_EX_ group, within‐participant repeated measures correlations were performed using the *rmcorr* package (version 0.3, Nick Golding, CRAN, The Netherlands) to assess the relationships between SA, mental effort, VA, and TSI responses (deltas from baseline across ICCT). This was performed to assess possible interdependences between variables considering the 5 time points per participant (∆20%, ∆40%, ∆60%, ∆80%, ∆TF from baseline). The coefficient provided by the correlations (*r*_rm_) was interpreted as *r*_rm_ < 0.1 indicating a very small effect, 0.1 ≤ *r*_rm_ < 0.2 a small effect, 0.2 ≤ *r*_rm_ < 0.3 a moderate effect, and *r*_rm_ ≥ 0.3 a large effect.

## 3 Results

### 3.1 ICCT Cycling Performance and HR Responses

The COG_EX_ group performed 7.0 ± 1.4 stages before reaching TF defined as a cycling effort ≥ 90/100. As shown in Table 1, the maximal cycling power at TF was 3.29 ± 0.51 watt·kg^−1^ with a HR_max_ of 175 ± 12 bpm (91.3% ± 6.4% HR_max_).

**TABLE 1:** Absolute values of cycling effort, percentage of maximal heart rate (%HR_max_), and absolute, body mass‐normalized, and relative power output recorded at 20%, 40%, 60%, 80%, and task failure (TF) of the total ICCT duration.

% total ICCT duration	Cycling effort (CR_100_)	%HR_max_	Absolute power output (W)	Normalized power output (W·kg^−1^)	Relative power output (% of TF)
20%	13.6 ± 8.4	56.2 ± 5.4	71 ± 10	0.98 ± 0.02	30.6 ± 5.0
40%	25.3 ± 11.4	64.5 ± 6.2	113 ± 17	1.56 ± 0.13	47.9 ± 3.8
60%	44.2 ± 12.7	76.7 ± 6.9	155 ± 26	2.14 ± 0.25	65.2 ± 2.5
80%	68.4 ± 11.0	83.9 ± 6.9	196 ± 36	2.72 ± 0.38	82.6 ± 1.3
TF	92 ± 2.3	91.3 ± 6.4	238 ± 46	3.29 ± 0.51	100 ± 0

### 3.2 Sustained Attention Performance

**FIGURE 2:**
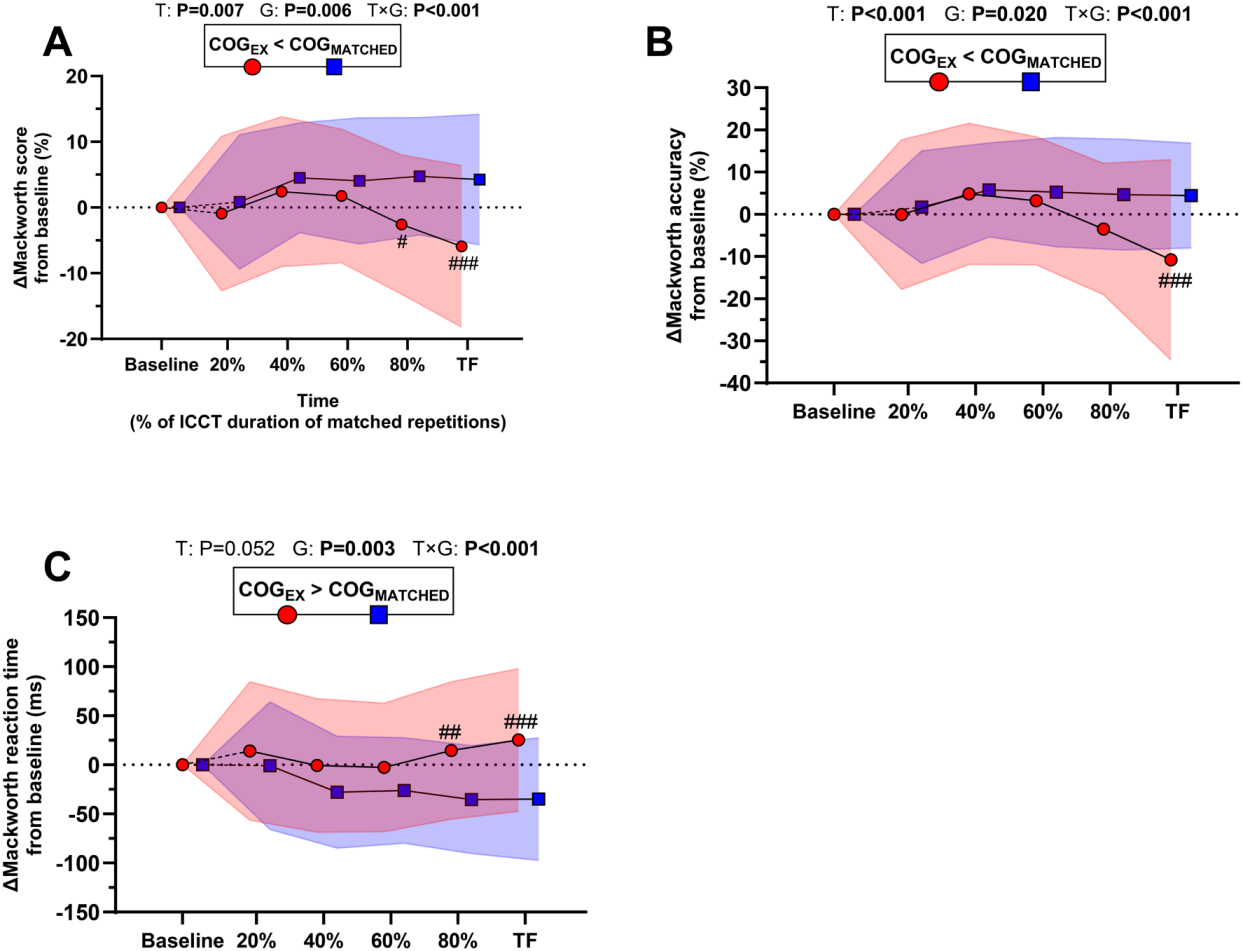
Global score (A), accuracy (B) and reaction time (C) from the Mackworth task performed either without exercise (COG_MATCHED_) or during ICCT (COG_EX_). Data are presented at baseline, 20%, 40%, 60%, 80% and task failure (TF) of the total ICCT duration, or at matched time‐points in the COG_MATCHED_ group. Data are presented as mean ± standard deviation (colored areas) for *N* = 40 in each group. *p* values for main effects of *Time* (T), *Group* (G), and *Time × Group* (T *×* G) are indicated on the panels. Post hoc significant differences for *Group* effect are shown in boxes above each graph. Post hoc significant differences for *Time × Group* interaction are marked with the following symbols: # (##, ###): *p* value < 0.05 (< 0.01, < 0.001) vs. respective COG_MATCHED_ time‐point.

As a reminder, cognitive assessments have been performed in a matched way in both COG_EX_ and COG_MATCHED_ groups. For the Mackworth score, main effects of *time* (*F*_5,390_ = 3.6, *p* = 0.005, $\eta_{p}^{2}$ = 0.044), *group* (*F*_1,78_ = 17.1, *p* < 0.001, $\eta_{p}^{2}$ = 0.18), and *time* × *group* interaction (*F*_5,390_ = 6.1, *p* < 0.001, $\eta_{p}^{2}$ = 0.072) were reported (Figure 2A). The Mackworth score was lower at 80%TF (−8.3 ± 5.7, *p* = 0.026) and TF (−11.0 ± 6.7, *p* < 0.001) in COG_EX_ compared to COG_MATCHED_. For Mackworth accuracy, main effects of *time* (*F*_5,390_ = 5.6, *p* < 0.001, $\eta_{p}^{2}$ = 0.067), *group* (*F*_1,78_ = 5.6, *p* = 0.020, $\eta_{p}^{2}$ = 0.068), and *time* × *group* interaction (*F*_5,390_ = 5.9, *p* < 0.001, $\eta_{p}^{2}$ = 0.071) were reported (Figure 2B). Mackworth accuracy was lower at TF (−17.1 ± 6.8, *p* < 0.001) in COG_EX_ compared to COG_MATCHED_. For Mackworth reaction time, main effects of *time* (*F*_5,390_ = 2.4, *p* = 0.045, $\eta_{p}^{2}$ = 0.030), *group* (*F*_1,78_ = 9.4, *p* = 0.003, $\eta_{p}^{2}$ = 0.11), and *time* × *group* interaction (*F*_5,390_ = 6.2, *p* < 0.001, $\eta_{p}^{2}$ = 0.074) were reported (Figure 2C). Mackworth reaction time was increased at 80%TF (+58.2 ± 29.4 ms, *p* = 0.007) and TF (+68.1 ± 38.6 ms, *p* < 0.001) in COG_EX_ compared to COG_MATCHED_.

### 3.3 Neuromuscular Function Responses

**FIGURE 3:**
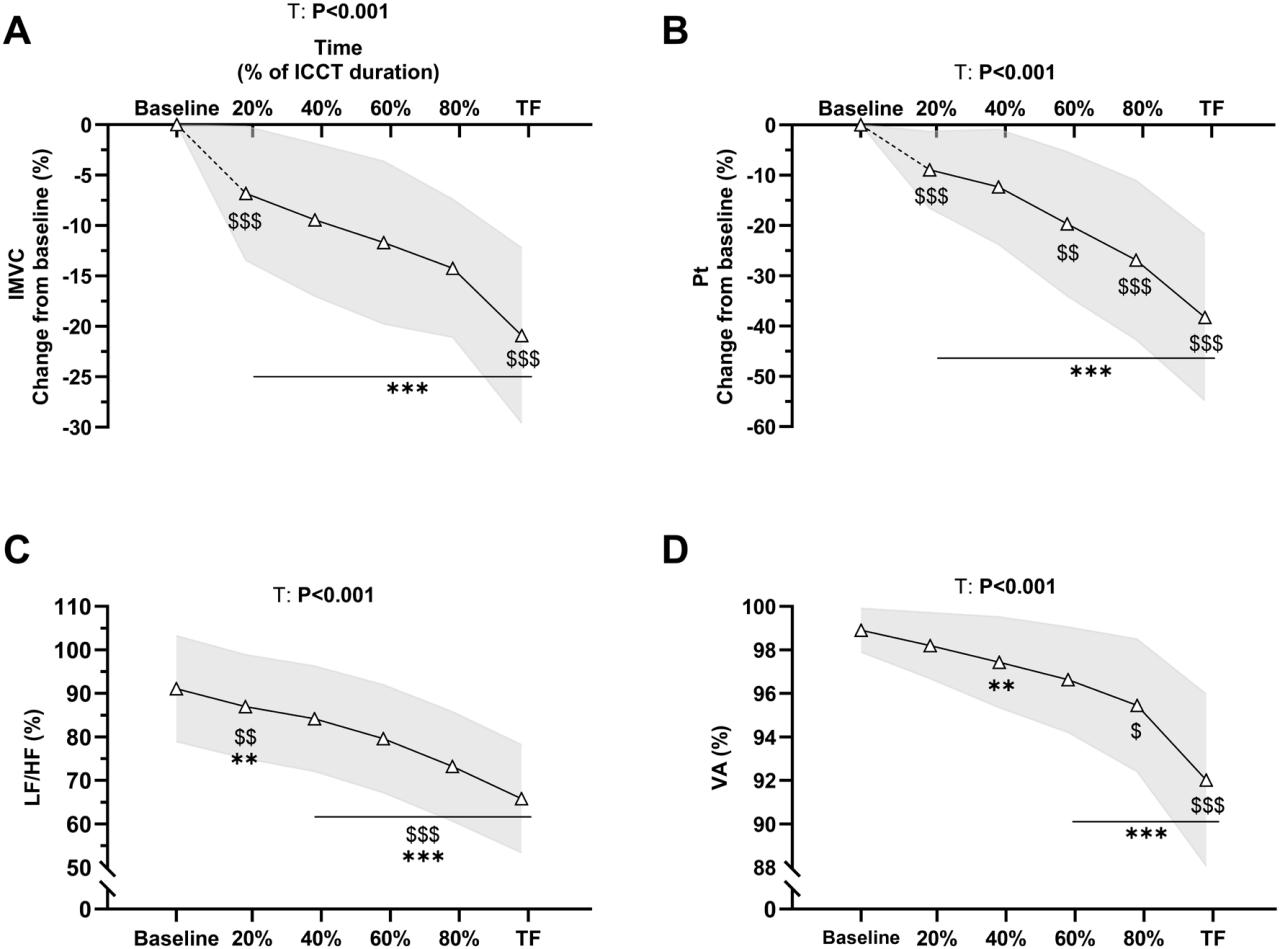
Changes in isometric maximal voluntary contraction (IMVC, A), peak twitch (Pt, B), low‐ to high‐frequency ratio (LF/HF, C) and voluntary activation (VA, D), throughout ICCT. Data are presented at baseline, 20%, 40%, 60%, 80% and task failure (TF) of total ICCT duration. Data are presented as mean ± standard deviation (gray area) for *N* = 40. *p* values for main effects of Time (T) are indicated on the panels. Post hoc significant differences for *Time* effect are marked with the following symbols: ** (***): *p* value < 0.01 (< 0.001) vs. Baseline. $ ($$, $$$): *p* value < 0.05 (< 0.01, < 0.001) vs. previous time‐point.

Regarding IMVC assessed during the ICCT on the COG_EX_ group exclusively, a main *time* effect was reported (*F*_4,133_ = 64.7, *p* < 0.001, $\eta_{p}^{2}$ = 0.63; Figure 3A). IMVC was reduced from 20%TF (*p* < 0.001 between baseline and 20%); the decrease was linear up to 80%TF (i.e., all *p* > 0.05), and IMVC was further decreased between 80%TF and TF (−6.7% ± 4.5%, *p* < 0.001). For peripheral indices of fatigue, a main effect of *time* was reported for LF/HF (*F*_4,123_ = 88.6, *p* < 0.001, $\eta_{p}^{2}$ = 0.71; Figure 3B) and Pt changes (*F*_4,123_ = 87.7, *p* < 0.001, $\eta_{p}^{2}$ = 0.71; Figure 3C). LF/HF and Pt changes were lower at 20%TF compared to baseline (*p* = 0.003 and *p* < 0.001, respectively) and were further decreased at 80%TF and TF (−7.4% ± 7.6%, *p* < 0.001 and −11.4% ± 9.8%, *p* < 0.001, respectively). For VA as a central index of fatigue, a main effect of time was reported (*F*_4,125_ = 50.9, *p* < 0.001, $\eta_{p}^{2}$ = 0.59; Figure 3D). VA was lower at 40%TF compared to baseline (*p* = 0.003) and was further decreased between 60% to 80%TF (−1.2% ± 0.8%, *p* = 0.03) and between 80%TF and TF (−3.5% ± 1.3%, *p* < 0.001).

### 3.4 PFC Oxygenation Responses

**FIGURE 4:**
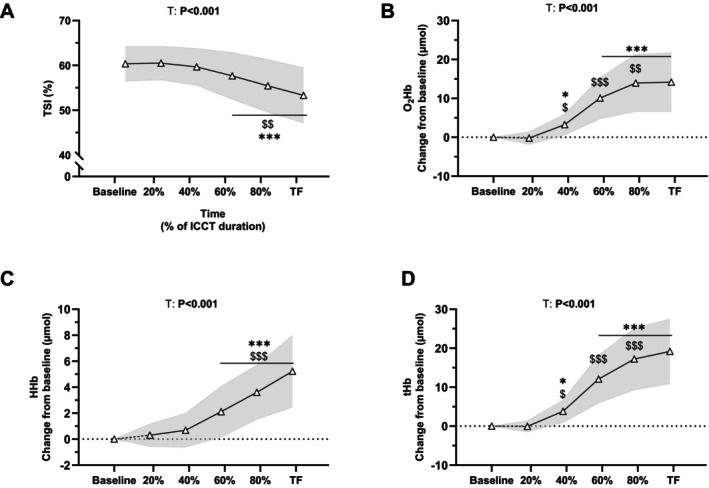
Prefrontal cortex oxygen tissue saturation index (TSI, A) and changes in NIRS‐derived indices (oxyhaemoglobin—O_2_Hb; deoxyhaemoglobin—HHb; total hemoglobin—tHb, B–D) throughout ICCT. Data are presented at baseline, 20%, 40%, 60%, 80% and task failure (TF) of the total ICCT duration. Data are presented as mean ± standard deviation (gray areas) for *N* = 40. *p* values for main effects of *Time* (T) are indicated on the panels. Post hoc significant differences for *Time* effect are marked with the following symbols: * (***): *p* value < 0.05 (< 0.001) vs. Baseline. $$ ($$$): *p* value < 0.01 (< 0.001) vs. previous time‐point.

For prefrontal TSI during ICCT, a main effect of *time* was observed (*F*_2,53_ = 48.4, *p* < 0.001, $\eta_{p}^{2}$ = 0.57; Figure 4A). TSI was decreased at 60%TF compared to baseline (*p* < 0.001) and was further decreased at 80%TF (−2.4% ± 1.2%, *p* < 0.001) and between 80%TF and TF (−2.1% ± 1.4%, *p* = 0.002). A main effect of *time* was observed for prefrontal O_2_Hb (*F*_2,53_ = 88.3, *p* < 0.001, $\eta_{p}^{2}$ = 0.75), HHb (*F*_2,53_ = 94.8, *p* < 0.001, $\eta_{p}^{2}$ = 0.76) and tHb (*F*_2,53_ = 118, *p* < 0.001, $\eta_{p}^{2}$ = 0.80). O_2_Hb and tHb (Figure 4B,D) were increased at 40%TF compared to baseline (*p* = 0.032 and *p* = 0.013, respectively) and were further increased at 60%TF (+6.8 ± 2.3 μmol, *p* < 0.001, +8.8 ± 3.2 μmol, *p* < 0.001, respectively) and at 80%TF (+3.7 ± 1.8 μmol, *p* = 0.005, +5.1 ± 2.6 μmol, *p* < 0.001, respectively) before plateauing between 80%TF and TF (both *p* = 1.0). HHb (Figure 4C) was increased at 60%TF compared to baseline (*p* < 0.001) and was further increased at 80%TF (+1.4 ± 0.7 μmol, *p* < 0.001) and between 80%TF and TF (+1.7 ± 0.8 μmol, *p* < 0.001).

### 3.5 Perceived Cycling Effort and Mental Effort Responses

**FIGURE 5:**
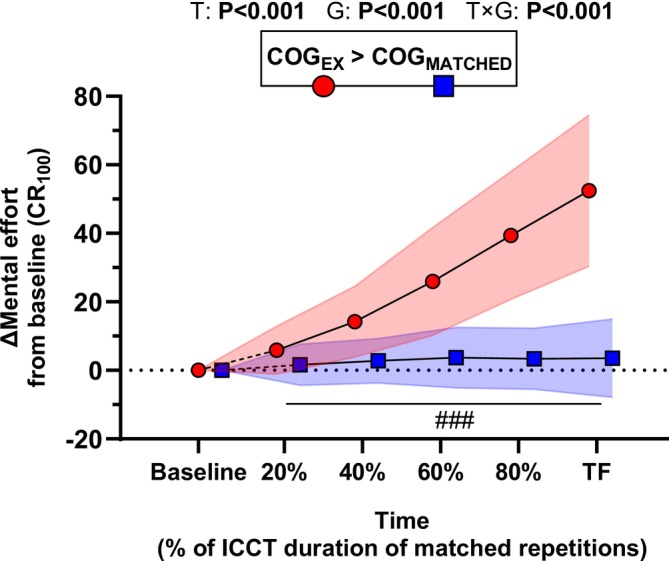
Mental effort associated with performing the Mackworth task, repeated without exercise (COG_MATCHED_) and during the ICCT (COG_EX_). Data are presented at baseline, 20%, 40%, 60%, 80% and task failure (TF) of the total ICCT duration, or at matched time‐points in the COG_MATCHED_ group. Data are presented as mean ± standard deviation (colored areas) for *N* = 40 in each group. *p* values for main effects of *Time* (T), *Group* (G), and *Time* × *Group* (T × G) are indicated on the panels. Post hoc significant differences for *Group* effect are shown in boxes above each graph. Post hoc significant differences for *Time × Group* interaction are marked with the following symbols: ###*p* value < 0.001 vs. respective COG_MATCHED_ time‐point.

For the COG_EX_ group, the cycling effort increased linearly across the ICCT (*F*_4,156_ = 818, *p* < 0.001, $\eta_{p}^{2}$ = 0.96) up to TF criteria (92 ± 2.3 on the CR_100_; Table 1). For mental effort, main effects of *time* (*F*_5,390_ = 133, *p* < 0.001, $\eta_{p}^{2}$ = 0.63), *group* (*F*_1,78_ = 109, *p* < 0.001, $\eta_{p}^{2}$ = 0.58), and *time* × *group* interaction (*F*_5,390_ = 106, *p* < 0.001, $\eta_{p}^{2}$ = 0.057) were reported (Figure 5). Mental effort was higher from 40%TF (+11.4 ± 6.2, *p* = 0.001) to TF (+48.9 ± 10.3, *p* < 0.001) in COG_EX_ compared to COG_MATCHED_.

### 3.6 Repeated Measures Correlations Between SA, Mental Effort, VA, and TSI

**FIGURE 6:**
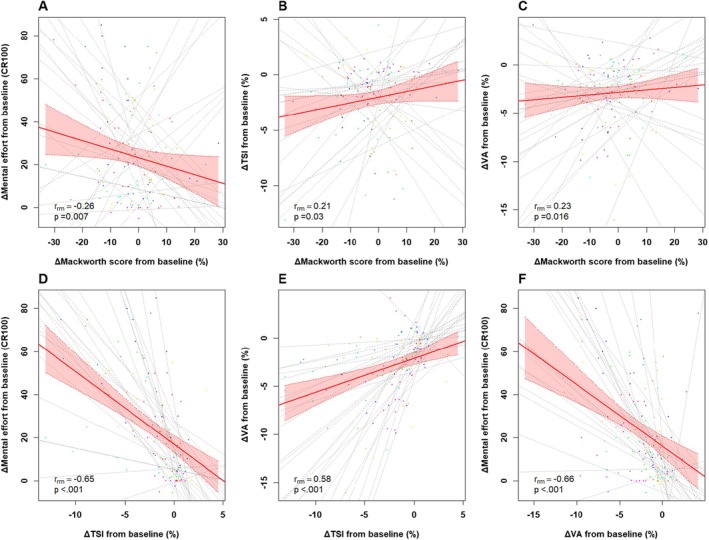
Repeated measures correlations at 20%, 40%, 60%, 80% and task‐failure (TF) of the total ICCT duration for variables expressed as delta from baseline. The correlated variables are sustained attention (ΔMackworth score) with mental effort (ΔMental effort, A), PFC oxygenation (ΔTSI, B), and voluntary activation (ΔVA, C), as well as ΔTSI with ΔMental effort (D), ΔVA (E), and finally ΔVA with ΔMental effort (F). Data points represent individual trials, with points of the same color corresponding to the same participant. Dashed lines indicate individual regression lines, while the solid red line shows the overall repeated measures correlation for *N* = 40 (COG_EX_ group). The shaded red area represents the 95% confidence interval. Each subfigure includes the repeated measures correlation coefficient (*r*_rm_), and the associated *p* value, calculated using the *rmcorr* package.

Repeated‐measures correlations between ∆SA, ∆mental effort, ∆VA, and ∆TSI across the ICCT showed that lower SA (Mackworth score) was associated with greater cerebral deoxygenation (TSI: *r*_rm_ = 0.30 [95% CI: 0.15, 0.43], *p* < 0.001), lower voluntary activation (VA: *r*_rm_ = 0.18 [0.03, 0.33], *p* = 0.02), and higher perceived effort (mental effort: r_rm_ = −0.32 [−0.45, −0.17], *p* < 0.001) (Figure 6A–C). TSI also correlated negatively with mental effort (*r*_rm_ = −0.75 [−0.81, −0.68], *p* < 0.001) and positively with VA (*r*_rm_ = 0.58 [0.47, 0.68], *p* < 0.001) (Figure 6D,E). Finally, VA correlated negatively with mental effort (*r*_rm_ = −0.60 [−0.69, −0.49], *p* < 0.001), suggesting that greater central fatigue was associated with higher subjective effort (Figure 6F).

## 4 Discussion

The aim of this investigation was to explore simultaneous SA performance, PFC oxygenation, and neuromuscular fatigue etiology during a new incremental cognitivo‐motor exercise in trained participants. The main findings are that: (i) SA performance was not enhanced for low‐to‐moderate cycling intensities but was impaired for strong to extremely strong cycling efforts (≥ 80%TF, ≥ 84%HR_max_); (ii) significant PFC deoxygenation appeared for strong cycling effort (≥ 60%TF, ≥ 77%HR_max_) and was correlated with SA performance; (iii) central fatigue appeared early for moderate cycling effort (≥ 40%TF, ≥ 64%HR_max_) and was mainly correlated with PFC deoxygenation and mental effort.

### 4.1 SA Performance Did Not Follow an Inverted‐U Curve Across Incremental Cycling

To our knowledge, this is one of the first studies to examine SA across exercise intensities throughout an incremental cycling and cognitive test (*cf*. CMDT). Mackworth score was preserved until COG_EX_ participants reached a very strong (score −8.3% ± 5.7%) to extremely strong (score −11.0% ± 6.7%) cycling intensities (≥ 80%TF, ≥ 84%HR_max_). Our results are reinforced by the observations in the COG_MATCHED_ group for whom the performance along the Mackworth task was maintained from baseline (score of 63.6% ± 10.9%) to TF (score of 67.4% ± 10.4%), dismissing any learning effect or any mental fatigue effect with such a succession of Mackworth sequences. The unimproved SA performance during low‐to‐moderate exercise intensities contradicts our initial hypothesis, while its impairment near TF aligns with our expectations.

We challenged two main principles of the inverted U‐shaped theory [2, 29]. Firstly, for efforts rated as “low” (20%TF, 13.6 ± 8.4/CR_100_) to “moderate” (40%TF, 25.3 ± 11.4/CR_100_), SA performance was not statistically different from baseline values, suggesting that cycling at these intensities did not improve SA. While it has been suggested that physical activity increases arousal (e.g., noradrenaline secretion), improving cognition facilitated by neurotransmitter release, it is possible that SA performance remained unchanged in the present study for these cycling intensities owing to the timing of consideration of the so‐called dual‐task (i.e., during vs. post‐moderate cycling). Indeed, lower effect sizes related to the cognition modifications have been reported during CMDT compared to when the studied cognitive task was performed a few minutes postexercise [2]. While examining executive function tasks, Stone et al. did not find any cognitive improvement during an incremental running test performed by trained soldiers. This aligns with our results using a sustained attention task [30].

Secondly, contrary to what could have been expected, SA performance with cycling efforts rated as strong (60%TF, 44.2 ± 12.7/CR_100_) was not impaired in trained adults. We suggest that the fitness level is an important factor that likely explains this result, as less trained participants were shown to decrease their SA performance during “strong” cycling efforts [18]. A direct comparison between these two types of population would therefore be of interest to assess the impact of training level on our measurements. We observed a correlation between the Mackworth score and TSI (*r*_rm_ = 0.30), which suggests a potential association between PFC oxygenation and cognitive performance during CMDT. While this does not establish causality, the result is consistent with the hypothesis that neural resource allocation may play a role during task execution [10]. We may speculate that the increase in oxy‐ and total hemoglobin observed with NIRS up to 80%TF is a proxy of the likely efficient neurovascular coupling in the PFC, at least efficient enough to cope with the progressive task‐induced metabolic activity so that the SA performance can still be maintained for submaximal intensities. The observation of a decreased TSI associated with a plateau in oxy‐ and total hemoglobin under the prefrontal probe are signs that the neurovascular coupling is challenged in its ability to fully cope with the metabolic activity of the local cerebral networks above 80%TF. These findings underscore the importance of investigating the interactions between oxygenation profiles and SA performance during CMDT in populations that are more susceptible to cerebral deoxygenation during submaximal exercise or due to challenging environmental conditions (e.g., heat, hypoxia), where oxygen delivery to the prefrontal cortex may be suboptimal.

When participants rated cycling effort as very strong or higher (≥ 80%TF, 68.4 ± 11.0 on the CR_100_; 83.9 ± 6.9%HR_max_), we observed a noticeable decrease in SA performance. These results are consistent with those obtained in a previous study assessing executive function abilities in soldiers during incremental running [30]. In the latter, they reported cognition to be impaired for intensities ≥ 80%HR_reserve_. This suggests that high‐intensity physical exertion, which also demands significant cognitive resources, can reduce performance on concurrent cognitive tasks. While it remains unclear whether their larger effect size ($\eta_{p}^{2}$ = 0.59 compared to $\eta_{p}^{2}$ = 0.067 in the present study) stems from the cognitive domain examined (executive function vs. sustained attention) [14] and/or the modality of whole‐body exercise performed (running vs. cycling CMDT in the present study) [2], our study is the first to replicate such effects during cycling in a larger sample (*N* = 40). Additionally, our findings offer new insights by linking these effects not only to prefrontal cortex oxygenation kinetics, but also, novelly, to perceived mental effort and neuromuscular fatigue development. Our findings provide clear evidence of the deleterious impact of very intense exercise (≥ 87%HR_max_) on SA performance in trained adults, addressing the uncertainty noted by Browne et al. in their review [5].

### 4.2 CMDT Situation as a Potential Contributor to Central Fatigue

One of our hypotheses was the development of neuromuscular fatigue across ICCT, explaining in part a decrease in SA performance. Fatiguability appeared progressively from the beginning of the ICCT, as indicated by a linear decrease in IMVC (Baseline‐TF = −21% ± 9.2%). Comparable amounts of global fatigue were observed at the end of an incremental cycling test without a surimposed cognitive task, conducted up to volitional exhaustion (TF, ~187 bpm) using a very similar ergometer capable of rapidly assessing neuromuscular fatigue [31]. These findings suggest that the decrease in IMVC between CMDT and exercising alone was not different, but the etiology of fatigue remained scarcely reported [17, 18]. With the aim of clarifying the origin of the neuromuscular impairments during the ICCT, we investigated the kinetics of peripheral and central mechanisms of fatigue. Peripheral fatigue, as measured by Pt and LF/HF ratio, developed linearly along ICCT (−40.7% ± 15.1% and −25.2% ± 9.5%, respectively), which is consistent with the decline in IMVC. These decreases in Pt and LF/HF ratio are consistent with findings by Mira et al. who reported similar values under iso‐HR conditions near the end of a comparable incremental cycling protocol [at 92% HR_max_ (comparable to the TF condition in the present study): −44.5% ± 13.4% and −32.4% ± 14.6%, respectively] [31]. These similarities suggest that the surimposed cognitive task does not exacerbate the development of peripheral fatigue. Indeed, previous studies using repeated measures randomized designs with exercise protocols involving isolated leg exercises or cycling have found similar levels of peripheral fatigue between CMDT and physical exercise alone conditions and further support the notion that CMDT situations exert a more direct influence on motor command.

Voluntary activation tracked to estimate the central fatigue started to be significantly lower at 40%TF (−1.5% ± 0.6%) and was further depressed at TF (−7.0% ± 1.4%). The companion studies by Aboodarda and Mira et al. reported only modest impairments in VA under iso‐HR conditions “at 64% HR_max_, corresponding to the 40%TF condition in the present study: +0.9% ± 0.8%; and at 92% HR_max_ corresponding to the TF condition in the present study: –1.3% ± 0.6%, respectively” [31, 32]. Notably, these reductions were statistically significant only at the very end of the incremental cycling test without any superimposed cognitive task (≥ 96.2% HR_max_) [32]. The reported VA impairments with high intensities of cycling are traditionally explained by the increased release of metabolites, stimulating III/IV afference fibers known to project centrally and inhibit central command [33]. We suggest that in our case, the earlier onset and larger magnitude of central fatigue may, at least partially, reflect the influence of the concurrent cognitive task, which likely recruited additional brain resources. Recent studies suggested that central fatigue is increased at the end of challenging physical exercises performed with superimposed cognitive tasks (i.e., intermittent knee‐extensor contractions up to TF or 15‐min strong cycling exercise) [17, 18]. Thanks to a unique assessment design and setup, the present study adds evidence on the relevant VA perturbation on whole‐body CMDT where exercise intensity is incrementally manipulated. While causal relationships remain difficult to establish, repeated measure correlations underline the interdependence between VA decrement and cognitive performance (small correlation between Mackworth score and VA; *r*_rm_ = −0.18), which may be mediated by shared influences such as cerebral oxygenation changes and perceived mental effort.

The ICCT increased progressively the mental effort (Baseline‐TF = +52.2 ± 22.1/CR_100_) compared to the COG_MATCHED_ group who performed the SA tasks alone (+3.5 ± 4.2/CR_100_), suggesting that CMDT elicits significant mental fatigue compared to a cognitive task alone. It has been proposed that an increased mental effort originating through a complex nonautomated dual‐task paradigm can be associated with mental fatigue [34], and this may be detrimental for cognitive performance. Indeed, similar results were obtained by Chatain et al. who reported mental effort to be increased only in CMDT situations [17]. It is unlikely that mental fatigue directly inhibits the neural drive to muscles [35] but recent theoretical frameworks propose that it may be associated with increased brain metabolites such as glutamate or adenosine, possibly indirectly modulating the motor command [36, 37]. Other studies should explore the neuro‐metabolic substrates associated with mental fatigue in CMDT contexts to better understand its origin, as well as its direct and indirect effects on cognitive performance and neuromuscular control.

## 5 Limitations and Perspectives

In this study, we designed 3‐min CMDT stages to reflect ecologically valid, real‐world scenarios. However, we acknowledge that more research is needed to clarify the interaction between exercise intensity and duration, particularly as recent work has proposed a more holistic perspective on how exercise modulates cognitive performance. Rather than relying solely on a traditional intensity‐based framework, the concept of fatigue‐based neurocognitive modulation has emerged [38]. For instance, athletes may maintain cognitive performance during the initial stages of a constant‐load exercise, but experience declines toward the end due to psychophysiological drift induced by accumulating fatigue. This idea is supported by recent findings, which demonstrated that prolonged exercise can impair executive functions even in highly trained individuals [39].

Additionally, the characteristics of the Mackworth task used here, notably its repetitive structure and relatively automatized nature, may have contributed to the limited performance variation observed across moderate intensities. Although SA shares certain operational characteristics with selective and focused attention, it is generally regarded as a distinct cognitive process. The Mackworth clock task is hence commonly used as a proxy for SA performance. However, in the context of the present study, the broader nature of the task likely engages multiple attentional components, including the abilities to select, shift, and distribute attention. This broader attentional involvement should be acknowledged as a potential limitation when interpreting results solely in terms of SA.

One limitation of this study is that VA assessed from the peripheral nerve stimulation technique does not allow differentiating spinal from supraspinal mechanisms implicated in central fatigue. Future studies using, for instance, transcranial and cervicomedullar magnetic stimulation should be conducted to further explore supraspinal alteration in the cortico‐motor pathways as changes in cortical excitability and inhibition of the motor cortex during fatiguing whole‐body CMDT [40].

Some inherent limitations of NIRS must also be acknowledged. Due to its limited penetration depth, approximately half the interoptode distance, NIRS is primarily sensitive to cortical regions within the upper ~1 cm of the brain. Consequently, the technique may not capture deeper or more distributed changes in cerebral microcirculation, which can vary heterogeneously during exercise and cognitive tasks. The regional nature of the measurements restricts generalizability to the whole brain, or to other areas that may also be critical for complex motor and dual‐task performance. Future studies employing functional NIRS in whole‐body CMDT paradigms are warranted to further advance our understanding of cerebrovascular responses and to help address these limitations.

Finally, the motor dual‐task cost could not be evaluated due to the absence of a cycling‐only condition. Although several methodological parameters align with those employed by companion papers from Mira and Aboodarda et al. (e.g., supine position, increment duration, and proximity to task failure) [31, 32], allowing for meaningful contextualization of our findings, we acknowledge that a direct comparison, ideally using a paired or crossover design, would have provided clearer insights into the kinetics of neuromuscular fatigue induced specifically by the CMDT configuration. Due to ethical constraints through several peripheral nerve stimulation assessments, participants did not complete an additional experimental session; however, we remain confident that this limitation does not compromise the study's main conclusions.

Taken together, these findings offer valuable guidance for designing CMDT interventions in both athletic, professional, or clinical settings aiming to enhance the brain's capacity to manage competing demands [41, 42]. The extent to which sustained cognitive performance and resilience to central fatigue are trainable, particularly under high‐pressure CMDT conditions involving intense exercise or environmental stressors, remains an open question and a promising avenue for future research.

## 6 Conclusion

This study investigated the interplay between sustained attention (SA), prefrontal cortex (PFC) oxygenation, neuromuscular fatigue, and mental effort during a novel incremental cognitively demanding motor task (CMDT) in trained individuals. The findings challenge traditional interpretations of the inverted‐U hypothesis by showing no SA enhancement at low‐to‐moderate cycling intensities while SA was significantly deteriorated during very strong to maximal perceived efforts (≥ 80%TF; ≥ 84%HR_max_), coinciding with PFC deoxygenation and increased mental and physical fatigue.

Crucially, central fatigue emerged at moderate intensities and was closely associated with reductions in PFC oxygenation and heightened mental effort, suggesting a direct link between neural resource allocation, cerebral oxygenation, and motor output regulation.

## Author Contributions

T.G., M.H., P.H., and T.R. were involved in the design of the study. T.G. performed the data collection. T.G. drafted the first version of the manuscript, with all authors involved in subsequent revisions and approval of the final document.

## Conflicts of Interest

The authors declare no conflicts of interest.

## Supporting information


**Video S1:** Video excerpt from the Mackworth Clock Task. Green and red lights indicate correct and incorrect responses, respectively.

## Data Availability

The data that support the findings of this study are openly available in DTPERF at https://osf.io/s7vuz/files/osfstorage?view_only=5c79e9519b184a08b5849aec4abd5996.
